# Robust classification of wound healing stages in both mice and humans for acute and burn wounds based on transcriptomic data

**DOI:** 10.1186/s12859-023-05295-z

**Published:** 2023-04-25

**Authors:** Ksenia Zlobina, Eric Malekos, Han Chen, Marcella Gomez

**Affiliations:** University of California, Santa Cruz, CA USA

**Keywords:** Wound healing, Wound stage classification, Transcriptomics, Time-series expression

## Abstract

**Background:**

Wound healing involves careful coordination among various cell types carrying out unique or even multifaceted functions. The abstraction of this complex dynamic process into four primary wound stages is essential to the study of wound care for timing treatment and tracking wound progression. For example, a treatment that may promote healing in the inflammatory stage may prove detrimental in the proliferative stage. Additionally, the time scale of individual responses varies widely across and within the same species. Therefore, a robust method to assess wound stages can help advance translational work from animals to humans.

**Results:**

In this work, we present a data-driven model that robustly identifies the dominant wound healing stage using transcriptomic data from biopsies gathered from mouse and human wounds, both burn and surgical. A training dataset composed of publicly available transcriptomic arrays is used to derive 58 shared genes that are commonly differentially expressed. They are divided into 5 clusters based on temporal gene expression dynamics. The clusters represent a 5-dimensional parametric space containing the wound healing trajectory. We then create a mathematical classification algorithm in the 5-dimensional space and demonstrate that it can distinguish between the four stages of wound healing: hemostasis, inflammation, proliferation, and remodeling.

**Conclusions:**

In this work, we present an algorithm for wound stage detection based on gene expression. This work suggests that there are universal characteristics of gene expression in wound healing stages despite the seeming disparities across species and wounds. Our algorithm performs well for human and mouse wounds of both burn and surgical types. The algorithm has the potential to serve as a diagnostic tool that can advance precision wound care by providing a way of tracking wound healing progression with more accuracy and finer temporal resolution compared to visual indicators. This increases the potential for preventive action.

**Supplementary Information:**

The online version contains supplementary material available at 10.1186/s12859-023-05295-z.

## Background

Wound healing is a dynamic process consisting of 4 stages—hemostasis, inflammation, proliferation, and remodeling [1, 2]. Diagnosing the wound stage is important for developing appropriate treatment and improving wound care [3–5]. In practice, doctors monitor the progression of wounds through visual cues and adjust treatment in correspondence with an individual's response. However, waiting for visual cues may result in missed windows of opportunity to affect change in the trajectory of the wound. Transcriptomic data has the potential to provide more timely information.

Transcriptomics of wound tissues is widely used for research purposes to investigate biological reactions [6–12]. Differentially expressed genes at specific wound healing stages have been identified in previous works [8, 9, 13–17], but the signatures vary among experiments. The discrepancy might result from differences in experimental conditions or differing bioinformatic approaches. Aiming to identify the sources of discrepancies is complicated by the fact that different time points are captured in different published datasets. However, a systematic comparison of wound healing transcriptomics between different wounds has shown that transcriptomic changes during wound healing demonstrate similar traits in diverse tissues (skin, muscles, internal organs, and nervous system) and species [18]. This meta-analysis was performed using the most significant gene ontology terms corresponding to the lists of genes highly expressed at different stages of wounding. The analysis performed by [18] indicates that at least some genes should demonstrate conserved expression patterns in different wounds. These genes might become clinical indicators of wound stages.

There are many challenges in pursuing a rigorous approach to assess the state of a wound and predicted outcomes. Data-based predictive models for wound diagnostics have been presented previously [19, 20]. In [19], the authors present a model to predict delayed healing based on medical records gathered from many patients and which includes many parameters. In [20], the composition of pro and anti-inflammatory macrophages is predicted based on gene expression signatures of wound tissue. The former approach requires big data availability, while the latter relies on a few genes, making it sensitive to missing a small number of genes. There are few publicly available wound transcriptomic datasets, and among them, time point measurements are inconsistent [16, 21–24]. To highlight this, Additional file 1: Table S1 lists several existing wound transcriptomic datasets with corresponding information, such as time points of transcriptomic biopsies. The alignment between datasets can be visualized in Additional file 1: Figure S1. Therefore, a transcriptomic-based approach to assess wound stage must work in the absence of big data and be robust to the timing of biopsies and to large variations in gene expression measurement.

Wound healing stages follow a prescribed pattern; thus, it is reasonable to expect that a model based on gene expression profiles in wound healing can be constructed with widespread applicability. Moreover, we hypothesized that we could identify genes that are differentially expressed at specific stages universally across different wounds. Due to a lack of time points, most datasets do not cover all wound healing stages. A suitable method, then, is to resort to collecting several datasets in one analysis to achieve a finer temporal resolution. We develop a method that can leverage multiple distinct datasets at once and is guaranteed to provide optimal results with respect to the available data independent of size. More specifically, we perform a comparative analysis of gene expression between several publicly available wound transcriptomic datasets from mice and humans. We search for genes that demonstrate similar expression dynamics in different wounds. Previous publications have represented time-dependent biological processes as trajectories in a multidimensional space of gene expression [25–27]. In this paper, we consider wound healing as a trajectory in gene expression space and build a model for wound stage prediction.

We considered 10 publicly available wound transcriptomic datasets listed in Additional file 1: Table S1. We note that the lack of longitudinal studies has presented the greatest challenge.

The criteria for dataset selection were:

- the samples are from non-treated skin wounds
- a sample from non-wounded skin exists (zero-point for reference)
- at least three additional time points after wounding are included—presumably covering different stages of wound healing.

Only five datasets satisfy these requirements. The first three of these datasets are used for model training and the latter two for testing. These datasets include mouse surgical and burn wounds and human laceration and burn wounds.

This work aims to develop a tool to expand the use of transcriptomic data in the clinical setting for translational work. The model presented in this paper is the first transcriptomic-based tool proposed for distinguishing between the four traditional wound healing stages and can serve as a diagnostic tool in clinical settings. Real-time analysis of the wound stage is necessary to achieve a timely and customized treatment. Furthermore, this work can be leveraged to customize wound treatment by facilitating a sense-and-respond strategy for a feedback control systems approach to wound care [28]. It has been proposed that feedback control can be actively implemented as part of an automated healthcare system for precision medicine [29]. This complements current work around the Internet of things (IoT) in Healthcare [30, 31]. In the future, the performance of the model may be improved as additional wound gene expression data becomes available.

## Results

### Wound stage detection algorithm outline

**Fig. 1:**
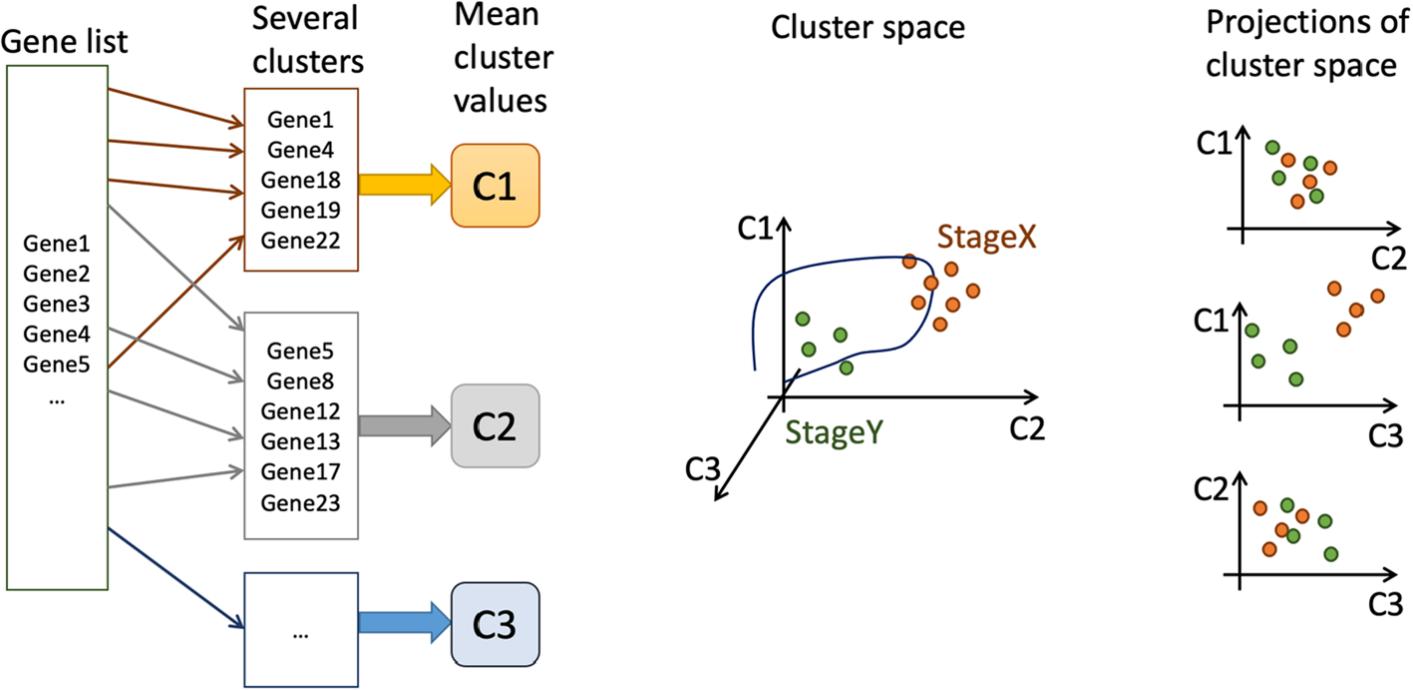
The pipeline of wound healing stage detection by transcriptomic data. From a list of all measured genes ("Gene list"), only differentially expressed genes are selected and grouped into "clusters" according to expression dynamics. The average gene expression value within each cluster at any given time is referred to as a "mean cluster value.” In the schematic, there are three such clusters (C1, C2, C3), and wound healing is presented as a temporal trajectory in the "cluster space". Each colored circle represents a three-dimensional vector in the cluster space corresponding to a single time point from a transcriptomic times series. The regions of the cluster space can then be classified into stages. This analysis may include considering several projections of the three-dimensional cluster space on to a two-dimensional plane to find the best one for discriminating between wound stages

The outline of our approach to wound stage detection is shown in Fig. 1. We first reduced the gene space to only those genes that are highly differentially expressed at any given time point across the three training datasets, inclusive of mouse surgical wounds, mouse burn wounds, and human skin wounds. The genes were then clustered into groups based on their temporal gene expression dynamics. Thus, data dimensionality can be reduced. Instead of preserving individual gene expression as states, expression of dozens of genes with similar dynamics are grouped. The wound healing process can be described by the temporal evolution of the mean value gene expression over all genes within each cluster. This allows us to represent the wound stage at any given time by the relative expression of the mean cluster values with respect to each other. Figure 1 presents a demonstrative example where temporal dynamics are grouped into three clusters.

The “mean cluster values” (C1, C2, C3,…) represent a space wherein the wound healing trajectory takes its course from wound onset to wound closure. Our task is to partition the n-dimensional space (where n is the number of clusters) into four regions that define each of the four wound healing stages (i.e., mathematically define the wound healing stages and trajectory). Our model should take as an input wound biopsy gene expression data, map the data to a point in this space, and, thereby, identify the wound healing stage that corresponds to our point of interest. To complete this task, we applied mathematical discrimination procedures to the space of “mean cluster values" as shown in Fig. 1. Two-dimensional projections are shown in Fig. 1, which aid in visualizing separatrices partitioning transcriptomic data from different wound stages.

Wound stages are not always defined in the description of the dataset analyzed; thus, we make careful assignments of the wound stage to each transcriptomic biopsy based on contextual information. In the figures that follow, all available data points across all time points are depicted in the projections of the cluster space. We train a machine learning (ML) classification model with a subset of the available data points and test with the remaining data points. We leverage an ML model known as a Support Vector Machine (SVM), which guarantees optimality and is suitable for relatively small datasets.

### Selection of informative genes

We filtered the datasets as follows. Only genes with reliably repeating replicates were used for analysis. In addition, we required that the final set of predictive genes was present in all three training datasets. Because we used both humans and mice, we required that documented orthologous genes were expressed in both species (see "Methods, Filtering" for details). Only 1622 genes were present in all three training datasets after filtering.

Next, we identified highly differentially expressed genes. Only 58 genes qualified as candidates for wound healing stage indicators (see "Methods. Choosing the set of characteristic genes"). We note that the final set of differentially expressed genes was selected in a highly conservative fashion to ensure robustness.

**Fig. 2:**
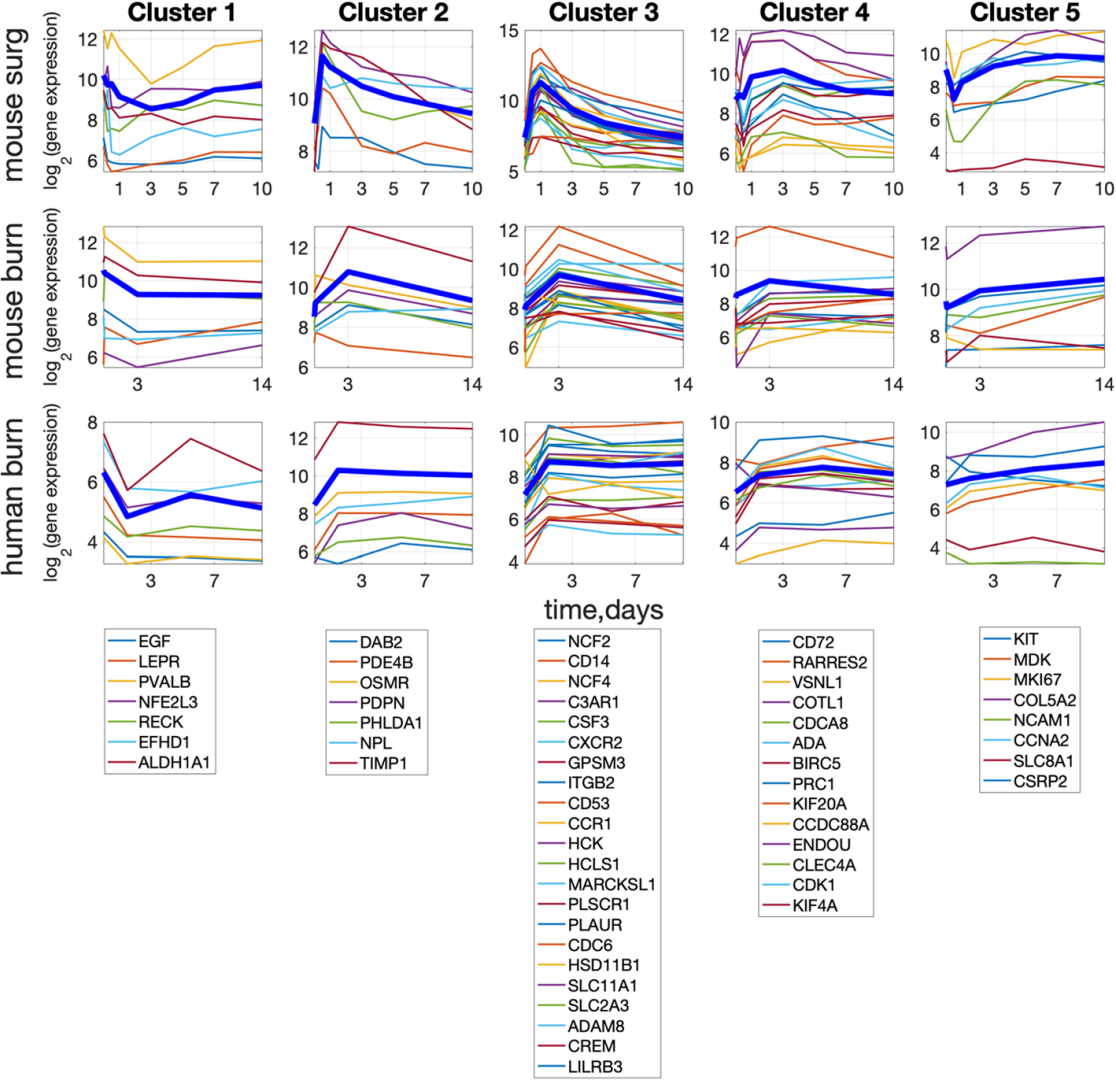
Gene expression dynamics of common highly expressed genes in the three datasets. Each row represents the same dataset, and each column represents the same cluster of genes listed in the legend under each column. The bold blue line in each plot corresponds to the mean value of gene intensity within each cluster (calculated for each dataset separately). Vertical axis: log2(Intensity), horizontal axis—time in days

The genes were divided into 5 clusters based on their expression dynamics during wound healing (see “Methods, Clustering” and Additional file 3: Table S3). The plots of gene expression dynamics of the resulting set of clustered genes are shown in Fig. 2.

We generally observe similar gene expression dynamics in each cluster. In contrast, the clusters exhibit distinct qualitative behavior, as can be seen by comparing images in the same row. Comparing images within the same column highlights the similarities and differences in gene expression dynamics across species and wound types. The genes of the first cluster are downregulated from 0 to 72 h in all datasets, and the genes of the third cluster are mainly upregulated at the same interval. However, if we compare the last cluster of genes, we see early downregulation of genes in the surgical wound that is not mirrored in either of the burn wounds. It is unknown whether the observed differences in this latter case are real or due to the low-resolution time steps masking the underlying gene expression dynamics. The lists of cluster genes and gene ontology analysis can be found in Additional file 3: Table S3.

**Fig. 3:**
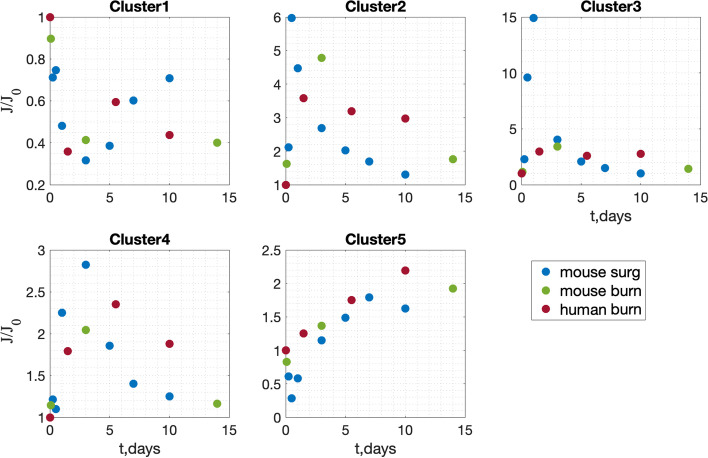
Normalized mean cluster value (J/J_0_) dynamics in three wound datasets. Clusters are indicated in Fig. 2

To finalize the comparison of gene expression between the wounds, we normalized the mean cluster value (see "Methods. Clustering"). In Fig. 3, normalized mean cluster value dynamics from the three datasets are plotted. One can see that all five normalized mean cluster gene expression values have very similar dynamics between wound datasets. Thus, the wound state at each time is represented by five numbers or by a point in 5-dimensional space. The wound follows a trajectory of healing in this 5-dimensional space.

After selecting the genes that will characterize the wound healing stage, we added two more wound gene expression datasets to the analysis: GSE28914 and GSE50425. Of the 58 genes identified above, 48 genes were found in GSE28914 and 40 genes in GSE50425. Thus, each cluster is sufficiently represented in the added test datasets, allowing the application of the mean cluster dynamics approach.

### Assignment of wound healing stages

In the datasets used for the model, wound healing stages are not rigorously defined, so there is some arbitrariness in assigning wound stages to specific time points in the data. Furthermore, wound healing stages are known to overlap. Therefore, we used gene expression intensity and timing to assign wound healing stages to the experimental data points.

The dataset GSE23006 contains the greatest number of time points, providing gene expression dynamics with the greatest detail. Many inflammatory genes in GSE23006 are upregulated at 12 and 24 h, represented by clusters 2 and 3, respectively (Fig. 2). We assume that time points 12 h and 24 h coincide with the peak of inflammation. Unfortunately, no other datasets under consideration contain these early time points and, similarly, high upregulation of these genes. We mark these time points as the "inflammation" stage.

All data points before “inflammation” are considered to be the “hemostasis" stage. They are 6 h in GSE23006 and 2 h in GSE460.

To distinguish between late and very late wound healing stages, presumably corresponding to proliferation and remodeling stages, we label the time points on days 3–7 as "proliferation" and those after day 7 as “remodeling”.

**Fig. 4:**
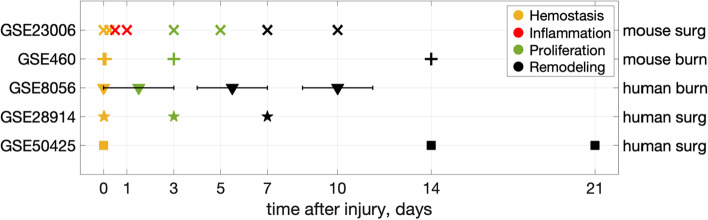
Map of wound healing stage assignment to publicly available datasets timepoints. Human dataset GSE8056 is represented by time intervals that are schematically shown with horizontal error bars

The complete map of assigned wound healing stages to data points is shown in Fig. 4. The assignments of wound healing stages to test datasets are required to assess the prediction accuracy of the ML model in the following section.

### Projections of cluster space

**Fig. 5:**
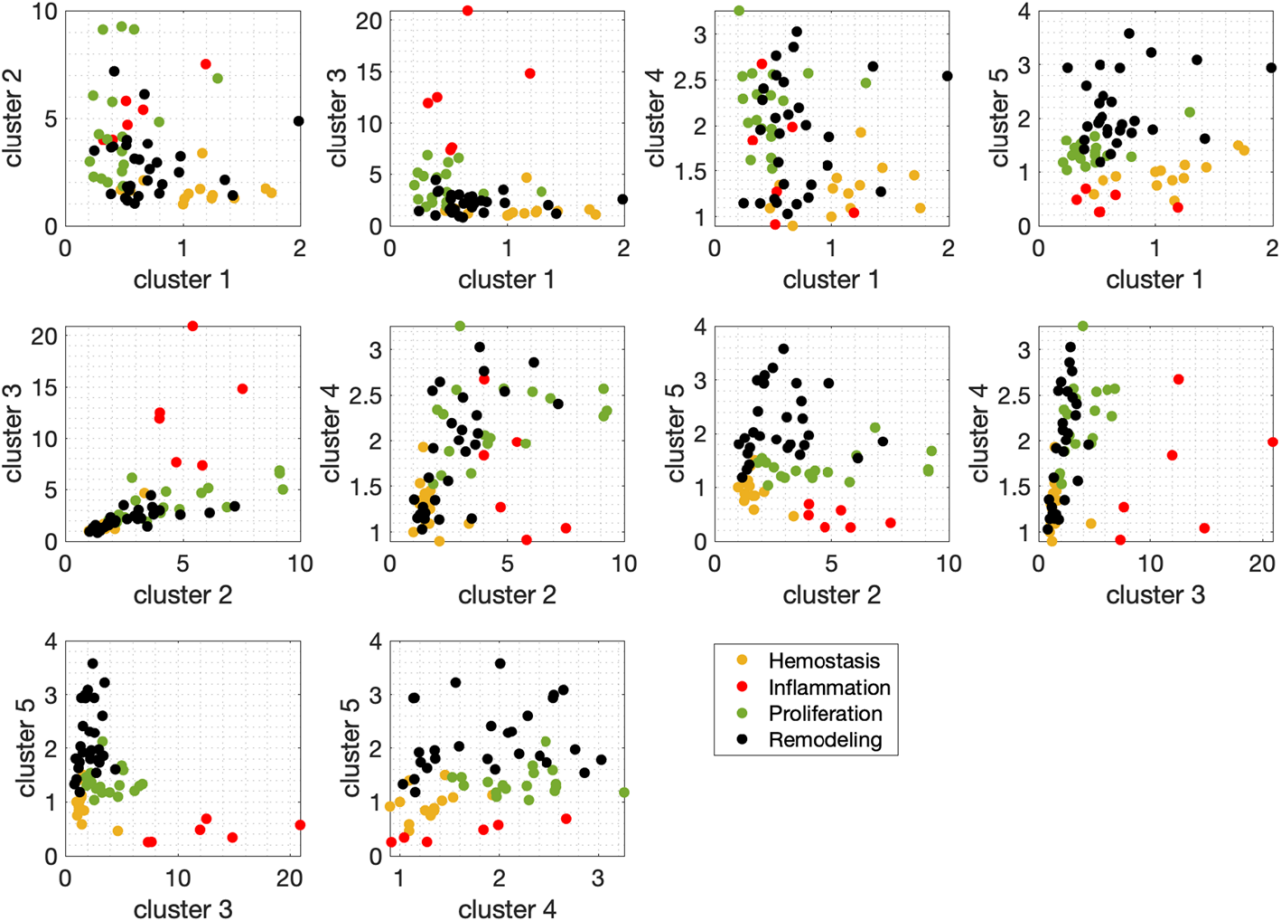
Projections of clusters onto two-dimensional spaces with data points, where the color corresponds to assigned wound healing stages. Both training and test data points are shown

Five “mean cluster values” for each wound biopsy represent a five-dimensional space where the wound healing trajectory is defined. In Fig. 5, experimental data points corresponding to biopsies from five datasets are shown in projections to 2-d planes. Different projections help to identify subspaces that aid in distinguishing wound healing stages. This is apparent when clouds of same-colored points are clearly separable. Then, classifying the stages of wound healing reduces to finding optimal boundaries between the clouds of points corresponding to different stages in the space of clusters shown in Fig. 5.

Consider Fig. 5 in detail. In the plane (cluster3, cluster4), inflammation points (red) are easily distinguishable from all others. In (cluster1, cluster5) the hemostasis points (yellow) are grouped apart from both proliferation and remodeling (green & black). The plane (cluster2, cluster5) is suitable for separating proliferation and remodeling stages (green and black points); however, a nonlinear curve is needed.

### A predictive model of wound healing stage detection

Despite the small number of time points available, we tested the possibility of creating a predictive ML model, specifically a multiclass classification model based on support vector machines (SVMs). Support vector machines classify data points by maximum-margin hyperplane, i.e., the hyperplane that has the largest distance to the nearest training-data points [32]. Classification algorithms based on SVMs guarantee optimal solutions, are one of the most robust prediction methods and can be adapted to handle nonlinear classifications [33].

One replicate from each time point in GSE23006, GSE460, and GSE8056 datasets served as training data. Wound healing stages were assigned to each point in the training dataset, as shown in the first three lines of Fig. 4. The remaining two replicates from those datasets (32 samples), along with the datasets GSE28914 and GSE50425, served as test data. These datasets contain data from different patients, so at each time point, several wounds are presented. There are 37 data points in the latter two datasets. The total number of sample points in the test dataset was 69.

The multiclass classification algorithm was organized as follows:

- **Step 1**: Linear SVM classification in the gene cluster subspace {Cluster 3, Cluster 4, Cluster 5} is performed to separate inflammation data points.
- **Step 2**: Polynomial SVM classification in {Cluster 1, Cluster 5} subspace is used to distinguish hemostasis data points from the rest (proliferation and remodeling).
- **Step 3**: Polynomial SVM classification in the subspace {Cluster 2, Cluster 5} is used to distinguish proliferation versus remodeling data points.

**Fig. 6:**
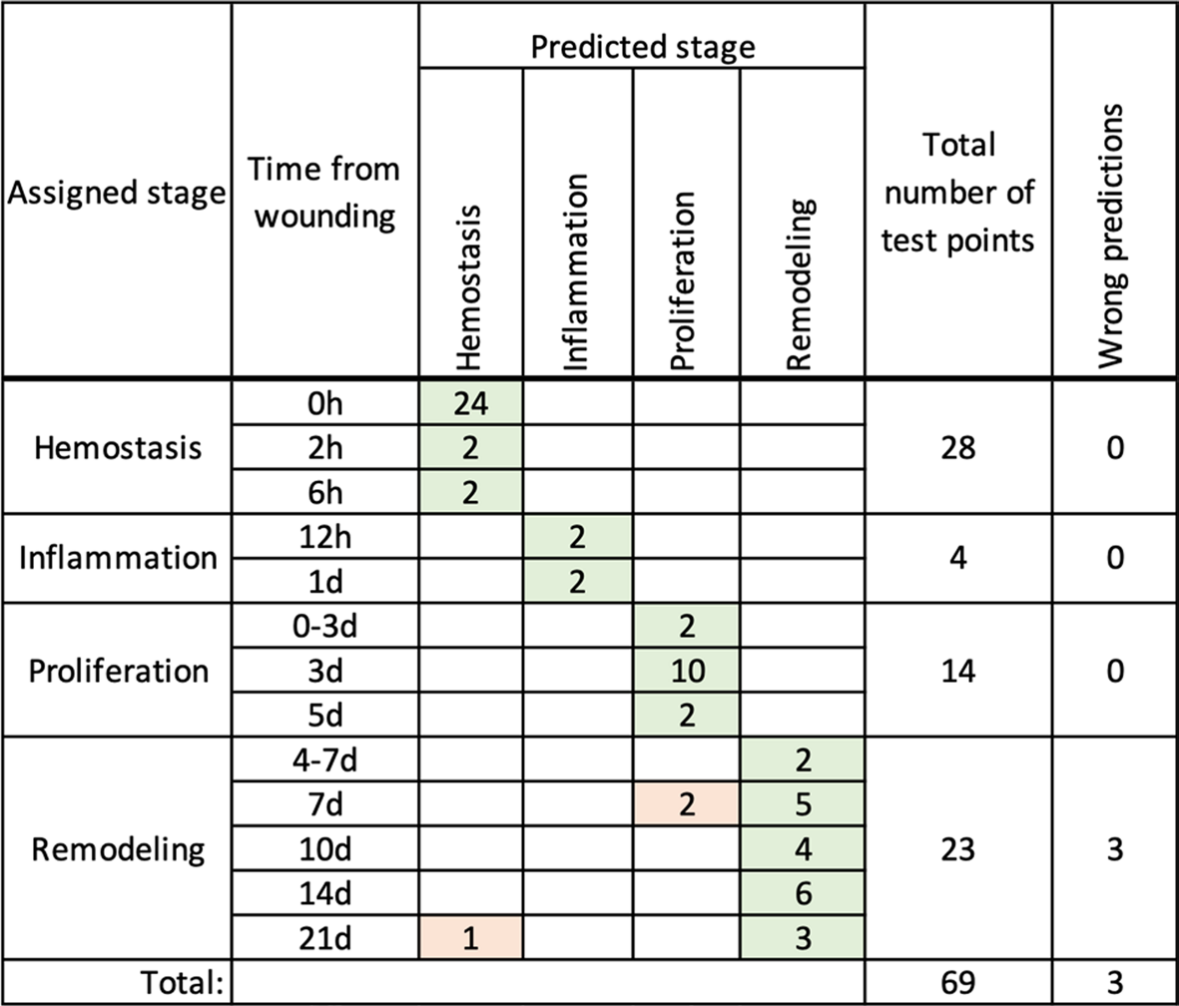
Wound healing stage prediction results. For each sampling time, the number of data points predicted to correspond with each stage is shown. The total number of tested data points and the number of erroneous predictions are in the last two columns. Green-shaded cells correspond to correct predictions and orange-shaded cells correspond to wrong predictions. Test data consists of replicates 2 and 3 from GSE23006, GSE460, GSE8056, and the full set of data points from GSE28914 and GSE50425

The prediction results are shown in Fig. 6.

Figure 6 shows that the model incorrectly predicted two data points from day 7, which were predicted to correspond to proliferation instead of remodeling. This is likely due to the model’s inability to discriminate the exact transition from proliferation to remodeling, given the ambiguity due to the natural overlap of wound healing stages. A second incorrect prediction is from day 21, where hemostasis is predicted instead of remodeling. This may be because the trajectory of wound healing is a loop that begins and ends in the same location of gene expression space—i.e., initial and end point transcriptomic profiles are highly similar.

Overall, in the described model setup, 66 of 69 data points are predicted as expected, in correspondence with manually assigned wound healing stages.

### Sensitivity analysis

We present sensitivity analysis to demonstrate the robustness of the model to missing data and show that the model is not overfitting.

#### Sensitivity to the training set

**Fig. 7:**
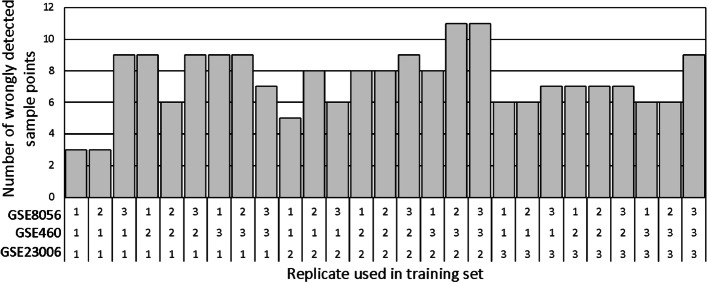
Model error dependence on the training set. One replicate from each of the datasets {GSE23006, GSE460, GSE8056} was used in training. The total number of training points remained 16, and the number of test points remained 69

As noted above, the datasets that have been used for training contain three replicates each, and only one replicate was selected for training, while the other two served as test data points. We varied the subset of data held for training and analyzed the performance of the model accordingly. Depending on which of the replicates is used for training, the performance of the predictive model varied, as shown in Fig. 7. However, for most of the test sets, there was no significant change in performance. The number of training points remained 16, while the number of test points was 69. In the worst-case scenario, the maximal number of wrong predictions was 11 points corresponding to a 16% error.

In many real situations, experimental data may contain errors or gene expression measurements may be missing. Our model is based on the expression of 58 genes but can still be used when a subset of the genes is missing in the data. The only “technical” requirement is—each cluster must be represented by at least one gene. We expected that fewer genes in each cluster would reduce the accuracy of wound stage prediction. We performed a sensitivity analysis of the model with respect to the number of available genes (see “Methods. Sensitivity analysis”). For this, we trained the model with the same set of genes and set of data points as described above, but the prediction on the test dataset was performed with a reduced subset of the 58 predictive genes. All data points were used in this test; that is, we applied the analysis to all timepoints from GSE23006, GSE460, GSE8056, GSE28914, and GSE50425.

**Fig. 8:**
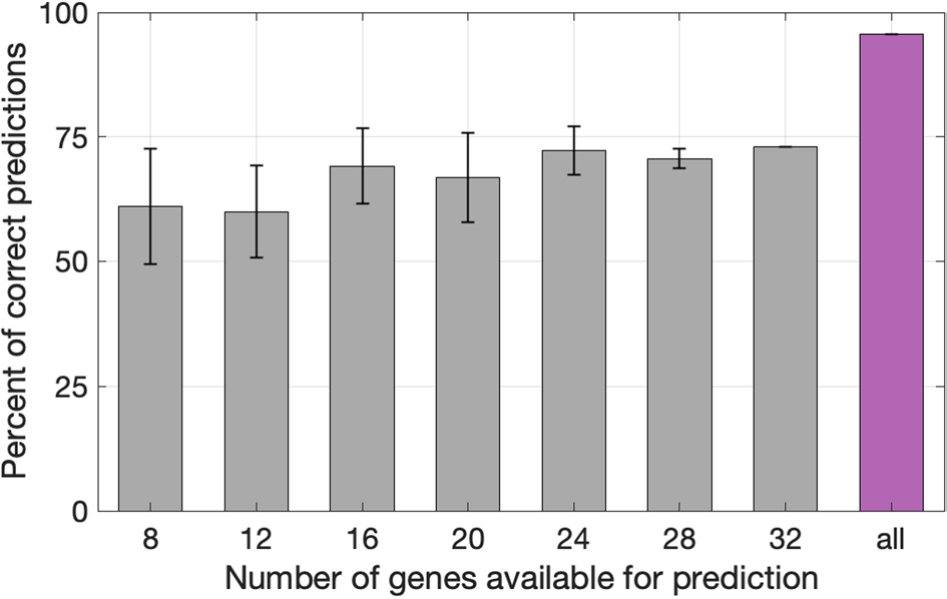
Model prediction sensitivity analysis with respect to missing genes. 20 random sets of N genes were selected from transcriptomic datasets (N = 8, 12, 16, 20, 24, 28). 85 wound biopsies were tested. The percentage of correctly predicted points was calculated for each set of genes. The resulting means and standard deviations are plotted. The magenta bar represents the original prediction percentage in 69 test points (shown in Fig. 6.) based on all available genes

The result of the sensitivity analysis is shown in Fig. 8.

### Complexity analysis

**Fig. 9:**
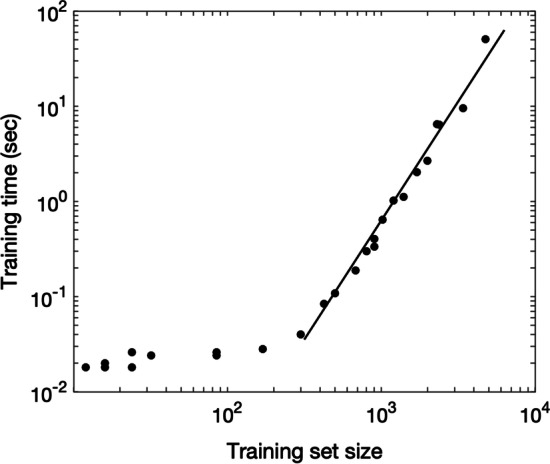
Complexity analysis: computer time required for the model training depends on the number of points in the training set. The original dataset is small, and big training sets were obtained by data augmentation. The slope shown by a straight line is 2.5

We did not encounter computational challenges, given that only 85 transcriptomic data points were available. However, we considered the applicability of our approach to a more extensive dataset. To estimate the training time of our algorithm in the case of larger datasets, we artificially created data points. New data points were generated by the multiplication of existing datapoint cluster values by a random number from the interval [0.8 1.2], i.e., by varying existing points within 20% of their original value. Training the algorithm five times for each training set size, we measured training time and then calculated mean time over five runs. The result is shown in Fig. 9. For datasets larger than 110 points, the dependence of training time on training set size is linear with a slope of 2.5 (log–log scale). Thus, for big datasets, the time complexity of our algorithms can be estimated as O(n^2.5^).

## Discussion

Investigation of wound healing is important not only for understanding but for improving treatment strategies and developing new-generation smart bandages for wound care.

Like many other biological processes, wound healing can be represented as a dynamic system that follows some prescribed trajectory. Here we consider wound healing in the multidimensional space of gene expression. For this, genes with a high fold-change of expression shared across various datasets were divided into five clusters based on expression dynamics. The average gene expression value in each cluster was used to represent the state of the wound as a point in a five-dimensional space. We hypothesized that such a representation could be used to predict the wound stage from gene expression measurements. A model was trained and tested on a relatively small amount of data due to limited publicly available longitudinal studies of acute wound healing. However, it gave promising results. It was shown that the wound stage can be predicted based on the level of expression of several indicative genes. Furthermore, averaging the genes within each cluster made the model robust to missing genes and experimental error as shown in the sensitivity analysis (see Fig. 9).

To investigate whether the gene clusters present any biological interpretations, we performed a gene ontology (GO) analysis of each cluster, see Additional file 3: Table S3 [34]. We note that the lists of genes were too short to reach statistical significance. However, the GO terms that emerged suggest that each cluster reasonably corresponds to a relevant biological process in wound healing from "inflammation" for cluster 3 to "cell development" for cluster 5. In the future, additional data can be used to map cluster dynamics to the biological processes of wound healing.

We note that our method utilizes non-injured skin biopsy for normalization. This means a single biopsy from a wound is insufficient for stage detection. The model requires the ratio of expression in the current wound state to non-injured skin. We cannot say if this is a consequence of variations in experimental setup or heterogeneity in the initial state of the skin [2, 35, 36]. With standardized data collection, we expect gene expression intensity rather than its ratio to healthy skin expression can be used in future models.

Our work supports the assumption that universal features of wound healing may be found even in different wound transcriptomic datasets (Fig. 3). Despite ongoing debates in the scientific literature about the applicability of the mouse skin wound model to humans [37, 38], we show similarities in both mouse and human data. We next consider the generalizability of the model to non-skin wounds. Several works compare the analysis of wounds in different tissues [14, 18, 39]. We tested our model on transcriptomic data from non-skin wounds. Our model cannot predict the wound healing stage in the rat cornea wound dataset GSE1001 or the mouse oral wound data from the GSE23006 dataset (results not shown).

The generation of new wound healing transcriptomics data may reveal that clusters suggested here can be improved to work on a broader range of wound conditions. Given the promising results of our technique across datasets, species, and wound types, we predict that our framework could serve as a basis for future models in different tissues. Even with the limited data available, we demonstrate that wound transcriptomic data can be utilized for future applications in medical diagnostics. Fine-tuning of gene clusters and additional control experiments will help to refine the robust wound stage prediction algorithm.

To our knowledge, this is the first attempt to create a clinically useful method for wound healing stage detection based on transcriptomic data. Of course, the development of a similar method for distinguishing chronic vs. acute wounds is of high practical interest [40]. In practice, wounds are defined as chronic based on the time passed without healing. However, if we can track wound healing trajectories, we may be able to identify early indicators of chronicity.

Collecting more wound transcriptomic data in the future will allow researchers to develop more accurate models for determining the stage of the wound. The success of our model on a relatively small gene set leads us to estimate that a minimal laboratory chip that measures only about 60 genes would be sufficient for high-accuracy wound-stage detection.

## Conclusions

Wound healing stage detection is of interest, as it can help to make decisions on treatment applications at the appropriate stage, and more than that, it can enhance the development of smart bandages for precision medicine.

In this work, a method for wound healing stage detection based on gene expression is presented. The algorithm is universal for human and mouse wounds of burn and surgical types. This approach can be applied in clinical settings. Further development of the algorithm is needed to facilitate clinical applicability and may bring about a new diagnostic tool that will help to improve precision wound care.

## Methods

### Data selection

We initially considered 10 publicly available wound transcriptomic datasets listed in Additional file 1: Table S1.

The criteria for dataset selection were:

- The samples are from non-treated skin wounds
- A sample from non-wounded skin exists (zero-point for reference)
- At least three additional time points after wounding are included, presumably covering different stages of wound healing.

Only 5 datasets satisfy these requirements: GSE23006, GSE460, GSE8056, GSE50425 and GSE28914.

The mouse wound transcriptomic dataset GSE23006 was originally created for skin and oral mucosa wound comparison [14]. In this work we are using GSE23006-skin data. The timepoints represented in the dataset are 0 h, 6 h, 12 h, 24 h, 3d, 5d, 7d, and 10 days after injury. The timepoints of the mouse burn wound dataset GSE460 are 0 h, 2 h, 3d, and 14 days after injury. Human burn wound dataset GSE8056 [15] is formed of time intervals instead of points,0d, 1-3d, 4-7d, and > 7 days. We used these three transcriptomic datasets for creating and training the machine learning (ML)-model for wound stage detection.

Our training dataset consists of 16 biopsies from the above three wound datasets—one replicate from each. We tested the model on the remaining replicates from the same datasets (32 sample points) and on two human skin wound datasets GSE28914 [11] (timepoints: 0 h before and after injury, 3^rd^ and 7^th^ day) and GSE50425 (0 h, 14d and 21 days after injury). Several patients' biopsies are presented at each time point. We considered each individual patient biopsy as a test point, thus having 37 individual biopsies from two test datasets. Thus, the test dataset consists of 69 data points.

### Data filtering

#### Orthologous genes between mouse and human

To compare particular genes between human and mouse, we found orthologs—homologous genes between species. All orthologous genes in mouse and human were matched by gene symbol to their homologene ID in *the Human and Mouse Homology Class report*. (Source: http://www.informatics.jax.org/homology.shtml).

The genes are considered for further analysis only if orthologs are found in all datasets under consideration.

### Filtering data of multiple genes in the same row

Although most probes in microarray transcriptomics exhibit a one-to-one mapping of probe-to-transcript, this is not always the case. Similarities between the nucleotide sequences of different genes can result in non-unique mappings, witnessed in the form "gene1//gene2//gene3" in the datasets. To avoid faulty comparisons, we simply removed these rows from analysis.

### Filtering data based on consistency of replicates

In all considered datasets each of n genes is presented as three replicates in m timepoints. We first denoted the timepoints by ${t}_{j}$ such that $j=0,\dots ,m-1$, where the unwounded state is associated with $j=0$. Let ${G}^{k}$ be an R^nxm^ matrix composed of m time series gene intensity measurements for k^th^ replicate of each of n genes. Let the average gene intensity across the three replicates be given by

$$G=\frac{{\sum }_{k=1}^{3}{G}^{k}}{3}\in {R}^{n\times m}$$

where the division operator is applied component wise. Then the percent relative error for each replicate is given by

$$S^{k} = \frac{{\left| {G^{k} - G} \right|}}{G} * 100 \in R^{n \times m} ,$$

with component wise operation in the division. Next, we want to find the average relative error for each time point across all genes and its standard deviation. This gives a sense of how much each replicate deviates from the average across the replicates independent of the gene. Let the matrix

$$S=\left[{S}^{1}{S}^{2}{S}^{3}\right]\in {R}^{3n\times 8}$$

be a matrix composed of the matrices ${S}^{k}$. Then we averaged across the columns such that we arrived at a row vector where each entry $i$ contains the average value across all elements in column $i$ of matrix $S$. We denoted this vector by ${\overrightarrow{r}}_{AVG}\in {R}^{1\times 8}$. Similarly, we computed the standard deviation across the columns of $S$ and denoted this vector by ${\overrightarrow{r}}_{STD}\in {R}^{1\times 8}$.

Then we computed the threshold for the maximum relative error, which determined which data was kept and which was discarded based on an acceptable value for the relative error. The threshold for each time point was chosen to be as follows

$${\overrightarrow{r}}_{Thres}={\overrightarrow{r}}_{AVG}+4{\overrightarrow{r}}_{STD}$$

where we took four standard deviations above the mean, inclusive of 99.98% percent of data assuming a normal distribution (Note that three standard deviations is inclusive of 99.72%). We found the maximum relative error across the three samples, where the new matrix ${S}_{max}=max\left({S}^{1},{S}^{2},{S}^{3}\right)\in {R}^{n\times m}$. The maximum was taken element-wise across the three matrices, that is ${S}_{max}\left(i,j\right)=max({S}^{1}(i,j),{S}^{2}(i,j),{S}^{3}(i,j)$) and made an element-wise comparison across each row

$${S}_{max,\left(i,:\right)}>{\overrightarrow{r}}_{Thres}$$

for $i=1:n$ and any time-series containing an extreme outlier in any of the replicates at any time point was removed from the dataset and, hence, the row removed from matrix $G$ contained the average intensity across the replicates. Note that we treated each timepoint individually since there may be different degrees of variability through the different wound healing stages. We denoted the new matrix $\widehat{G}$, which contains a subset of the rows of $G$, after discarding rows with high variability across replicates.

### Filtration of repeated measurements of the same gene in each dataset

Some genes are mentioned in the dataset several times (several repetitions or several transcripts). In addition, for some genes the repeated rows contain too different expression dynamics. To leave "one gene – one row" we filtered based on the correlation between repeated gene rows.

Denote the ith row of the matrix $\widehat{G}$ as ${\overrightarrow{g}}_{i}\in {R}^{1\times m}$. It contains time point mean intensity measurements of gene $i$ such that: ${\overrightarrow{g}}_{i}=\left[{g}_{i}\left({t}_{0}\right),{g}_{i}\left({t}_{1}\right),\cdots ,{g}_{i}\left({t}_{m}\right)\right]$. Suppose that there are $k$ vectors corresponding to one and the same gene:

$${\overrightarrow{g}}_{{i}_{1}}, ldots, {\overrightarrow{g}}_{{i}_{k}}$$

First, we found Pearson correlation coefficients between each pair of repeated gene intensities: ${C}_{nm}=corr\left({\overrightarrow{g}}_{n},{\overrightarrow{g}}_{m}\right),n\ne m$, we obtain ${k}^{2}-k$ correlation coefficients. The gene is kept for further analysis if at least two repetitions are highly correlated:

* $$\left({C}_{nm}\right)\ge C$$

In this work we used the threshold $C=0.9$. If the condition (*) was satisfied, we took one of the highly correlated gene intensity rows ${\overrightarrow{g}}_{n}$, ${\overrightarrow{g}}_{m}$ (we can take the mean between the intensities of two highly correlated genes). If the condition (*) was not satisfied, the gene was not included in further analysis.

### Several genes corresponding to the same homologene number

For some genes the same homologene identifier corresponded to two genes. For example, homologene identifier corresponding to gene X in mouse corresponded to genes X1 and X2 in human. In this case we checked if there was high correlation between genes X1 and X2 ($corr\left({\overrightarrow{g}}_{{X}_{1}},{\overrightarrow{g}}_{{X}_{2}}\right)>C$) and took one of them. Otherwise, these genes are not included in further analysis.

The number of genes left in each dataset at each filtering step is presented in Table 1. We emphasize that while we tried to come up with a standardized approach, other approaches can be considered.

**Table 1:** Numbers of genes in each dataset after each filtering step (described in Methods)

Dataset	GSE23006 mouse skin wound	GSE460 mouse burn skin wound	GSE8056 human skin wound
Initial number of genes	45,101	7275	54,675
After filtering multiple genes in same row	29,309	6130	31,762
After filtering based on consistency of replicates	26,519	5760	30,242
Unique gene names in filtered subset	13,626	4980	13,777
After filtering of repeated measurements of same gene in each dataset	8005	4449	9254
After filtering several genes corresponding to the same homologene number	7937	4441	9249

The 3 datasets contain 7937 (GSE23006), 4441 (GSE460) and 9249 (GSE8056) genes after filtering. The intersections contain even less genes, see Table 2.

**Table 2:** Number of genes in the intersections of each pair of datasets after filtration

Dataset 1	Dataset 2	N of common genes
GSE23006 mouse surg (7937)	GSE460 mouse burn (4441)	2441
GSE23006 mouse surg (7937)	GSE8056 human (9249)	5278
GSE460 mouse burn (4441)	GSE8056 human (9249)	2855

Intersection of all 3 datasets consists of 1622 genes

### Choosing the set of characteristic genes

Let $g\left(t\right)$ be intensity of gene expression at time t. Each transcriptomic microarray dataset contains gene expression in several timepoints: ${t}_{1}, {t}_{2},\dots$. For each gene in each dataset minimal and maximal expression may be defined as:

$${I}_{max}=max(g\left({t}_{i}\right) )$$

$${I}_{min}=min(g\left({t}_{i}\right) )$$

The maximum observed fold change is defined as:

$$\Delta G=\frac{{I}_{max}-{I}_{min}}{{I}_{min}}$$

For each dataset under consideration (GSE23006, GSE460, and GSE8056), the first 300 genes with the highest $\Delta G$ were selected. The comparative analysis of gene fold-change of the same genes in different datasets is provided in Additional file 2: Table S2. We selected the first 300 genes with the greatest fold-change in each of the three datasets. The intersection of these three subsets was 58 genes. Thus, 58 genes demonstrated high fold-change in all three datasets.

We note that many methods are considered in the literature to identify highly differentially expressed genes [41–46]. Those methods are well suited for identifying significant genes, associated pathways, and underlying biological processes. In this work, we are concerned with finding the largest set of genes consistently identified as differentially expressed across datasets. Our selection of the first 300 genes with the highest $\Delta G$ from each dataset provides a large set of genes to start with, but a small enough number to ensure confidence that genes are indeed differentially expressed.

Comparison of $\Delta G$ values between datasets and additional analysis are presented in Additional file 2: Table S2.

### Clustering

The set of selected 58 genes was divided into 5 clusters (see Fig. 2) based on the peak time in GSE23006 mouse surgical data, where peak time refers to the time window in which gene expression intensity is maximized. Cluster1 includes genes with peak time in mouse surgical wounds equal to 0–12 h, Cluster2–24 h, Cluster3–72 h, Cluster4–120 h, and Cluster5 > 120 h.

Next, the plots of the same genes in other datasets were divided into the same cluster as GSE23006 mouse surgical, independently of their expression dynamics in other datasets (Fig. 2).

To finalize the comparison of gene expression between the wounds, we normalized the mean cluster value by dividing by its initial value (t = 0, non-injured tissue):

$$Normalized\:mean\:cluster\:value=\frac{{J}_{i}}{{J}_{0}}$$

$${J}_{i}=\frac{1}{N}{\sum }_{k}^{N}{g}_{k}({t}_{i})$$

where the summation is over all genes within the cluster and N is the number of genes in the cluster (see Fig. 3).

### Sensitivity analysis

The model was trained with 16 data points from GSE23006, GSE460, and GSE8056, with mean cluster values calculated with 58 genes (Additional file 1: Table S3). The first replicate from each dataset was used for the model training. After that, the sensitivity of model predictiveness to the number of genes was performed.

For this, 20 random subsets of gene shortlists were selected. Each subset contained at least one gene from each cluster. Of all 58 genes used in our model, GSE28914 contains only 48 genes, and GSE50425 contained only 40 genes (see Additional file 2: Table S2 data table). The number of genes available in each of the five datasets is 32. The number of genes in the shortlists N was: 8 <  = N <  = 32.

All data points were used, including each replicate and each timepoint of five considered datasets (GSE23006, GSE460, GSE8056, GSE28914, and GSE50425)—85 data points.

The mean cluster value was calculated for each cluster based on the diminished number of cluster genes. The percent of timepoints for which the predicted wound stage coincided with the assigned wound healing stage was recorded for each random set of N genes. Mean value and standard deviation (STD) over 20 random choices were calculated and plotted in Fig. 7. For N < 32, all random subsets were different. For N = 32 only one subset exists (all genes existing in all five datasets), that's why STD = 0, Fig. 7).

The bar corresponding to the initial testing model is shown in magenta: with 69 timepoints, and using all available genes are shown: replicates 2 and 3 from GSE23006/GSE460/GSSE8056 (58 genes), GSE28914 (48 genes) and GSE50425 (40 genes).

### Software and online tools used for this work

Matlab 2020a, DAVID bioinformatics resources: https://david.ncifcrf.gov, Venny 2.1: https://bioinfogp.cnb.csic.es/tools/venny/.

## Supplementary Information

**Additional file 1.** Supplementary S1: “Data selection”—analysis of existing wound transcriptomic datasets. Supplementary S2: “Fold change as an indicator of highly differentially expressed genes”—comparison of 3 main datasets under consideration.

**Additional file 2.** An excel table of gene expression data used in the model.

**Additional file 3.** Matlab code with the model. To run the model the data file must be in the same folder.

## Data Availability

Publicly available datasets used in this work: GSE23006, GSE460, GSE8056, GSE28914 and GSE50425 from Gene Expression Omnibus database: https://www.ncbi.nlm.nih.gov/geo/. The filtered data created during the current study are available in Supplementary data file 2. Matlab code of the prediction model is available in Supplementary data file 3.

## References

[1] Gurtner G, Werner S, Barrandon Y (2008). Wound repair and regeneration. Nature.

[2] Canedo-Dorantes L, Canero-Ayala M, Skin acute wound healing: a comprehensive review. Int J Inflamm. 2019; article ID 3706315. 10.1155/2019/3706315 PMC6582859 31275545

[3] Krzyszczyk P, Schloss R, Palmer A, Berthiaume F (2018). The role of macrophages in acute and chronic wound healing and interventions to promote pro-wound healing phenotypes. Front Physiol.

[4] Zlobina K, Xue J, Gomez M (2022). Effective spatio-temporal regimes for wound treatment by way of macrophage polarization: a mathematical model. Front Appl Math Stat.

[5] Weigelt MA, Lev-Tov HA, Tomic-Canic M, Lee WD, Williams R, Strasfeld D, Kirsner RS, Herman IM (2022). Advanced wound diagnostics: toward transforming wound care into precision medicine. Adv Wound Care (New Rochelle)..

[6] Foster DS (2021). Integrated spatial multiomics reveals fibroblast fate during tissue repair. Proc Natl Acad Sci USA.

[7] Leon C (2020). Transcriptomic analysis of a diabetic skin-humanized mouse model dissects molecular pathways underlying the delayed wound healing response. Genes (Basel)..

[8] Roy S, Khanna S, Rink C, Biswas S, Sen CK (2008). Characterization of the acute temporal changes in excisional murine cutaneous wound inflammation by screening of the wound-edge transcriptome. Physiol Genomics.

[9] Wilkinson HN, Guinn BA, Hardman MJ (2021). Combined metallomics/transcriptomics profiling reveals a major role for metals in wound repair. Front Cell Dev Biol..

[10] Iglesias-Bartolome R, Uchiyama A, Molinolo A, et al. Transcriptional signature primes human oral mucosa for rapid wound healing. Sci Transl Med. 2018; 10(451). 10.1126/scitranslmed.aap8798 PMC6598699 30045979

[11] Nuutila K, Siltanen A, Peura M, Bizik J (2012). Human skin transcriptome during superficial cutaneous wound healing. Wound Repair Regen..

[12] Haensel D (2020). Defining epidermal basal cell states during skin homeostasis and wound healing using single-cell transcriptomics. Cell Rep.

[13] St Laurent G, 3rd, et al. Deep sequencing transcriptome analysis of murine wound healing: effects of a multicomponent, multitarget natural product therapy-Tr14. Front Mol Biosci. 2017;4:57. 10.3389/fmolb.2017.00057. 10.3389/fmolb.2017.00057 PMC5572416 28879183

[14] Chen L, Arbieva ZH, Guo S, Marucha PT, Mustoe TA, DiPietro LA (2010). Positional differences in the wound transcriptome of skin and oral mucosa. BMC Genom.

[15] Greco JA, Pollins AC, Boone BE, Levy SE (2010). A microarray analysis of temporal gene expression profiles in thermally injured human skin. Burns.

[16] Deonarine K, Panelli MC, Stashower ME, et al. Gene expression profiling of cutaneous wound healing. J Transl Med. 2007. 5:11 10.1186/1479-5876-5-11 PMC1804259 17313672

[17] Sato Y, Ohshima T (2000). The expression of mRNA of proinflammatory cytokines during skin wound healing in mice: a preliminary study for forensic wound age estimation (II). J Legal Med.

[18] Sass PA, Dabrowski M, Charzynska A, Sachadyn P (2017). Transcriptomic responses to wounding: meta-analysis of gene expression microarray data. BMC Genom.

[19] Jung K, Covington S, Sen CK, Januszyk M, Kirsner RS, Gurtner GC, Shah NH (2016). Rapid identification of slow healing wounds. Wound Repair Regen..

[20] Ferraro NM, Dampier W, Weingarten MS, Spiller KL (2017). Deconvolution of heterogeneous wound tissue samples into relative macrophage phenotype composition via models based on gene expression. Integr Biol.

[21] Ud-Din S, Wilgus TA, McGeorge DD, Bayat A (2021). Pre-emptive priming of human skin improves cutaneous scarring and is superior to immediate and delayed topical anti-scarring treatment post-wounding: a double-blind randomised placebo-controlled clinical trial. Pharmaceutics.

[22] Crompton RA, Williams H, Campbell L, Hui Kheng L, Saville C, Ansell DM, Reid A, Wong J, Vardy LA, Hardman MJ, Cruickshank SM. An epidermal-specific role for arginase1 during cutaneous wound repair. J Invest Dermatol. 2021; S0022-202X(21)02288-0. 10.1016/j.jid.2021.09.009 10.1016/j.jid.2021.09.009 34710388

[23] Kostarnoy AV, Gancheva PG, Lepenies B, Tukhvatulin AI, Dzharullaeva AS, Polyakov NB, Grumov DA, Egorova DA, Kulibin AY, Bobrov MA, Malolina EA, Zykin PA, Soloviev AI, Riabenko E, Maltseva DV, Sakharov DA, Tonevitsky AG, Verkhovskaya LV, Logunov DY, Naroditsky BS, Gintsburg AL (2017). Receptor Mincle promotes skin allergies and is capable of recognizing cholesterol sulfate. Proc Natl Acad Sci U S A.

[24] Kolumam G, Wu X, Lee WP, Hackney JA (2017). IL-22R ligands IL-20, IL-22, and IL-24 Promote wound healing in diabetic db/db mice. PLoS ONE.

[25] Rukhlenko OS, Halasz M, Rauch N (2022). Control of cell state transitions. Nature.

[26] La Manno G, Soldatov R, Zeisel A (2018). RNA velocity of single cells. Nature.

[27] Raychaudhuri S, Stuart JM, Altman RB, Principal component analysis to summarize microarray experiments: application to sporulation time series. Pac Symp Biocomput. 2000; 455–466. 10.1142/9789814447331_0043 PMC2669932 10902193

[28] Hosseini Jafari B (2021). A feedback control architecture for bioelectronic devices with applications to wound healing. J R Soc Interface.

[29] Zlobina K, Jafari M, Rolandi M, Gomez M. The role of machine learning in advancing precision medicine with feedback control. Cell Rep Phys Sci. 2022; Online Nov. 9. 10.1016/j.xcrp.2022.101149.

[30] Soufiene BO, Bahattab AA, Trad A, Youssef H. LSDA: Lightweight Secure Data Aggregation Scheme in Healthcare using IoT. In: Proceedings of the 10th International Conference on Information Systems and Technologies (ICIST '20). 2021; 22, 1–4. 10.1145/3447568.3448530.

[31] Soufiene BO, Bahattab AA, Trad A, Youssef H (2020). PEERP: an priority-based energy-efficient routing protocol for reliable data transmission in healthcare using the IoT. Procedia Comput Sci.

[32] Hastie T, Tibshirani R, Friedman J. The elements of statistical learning: data mining, inference, and prediction (second ed.). 2008; New York: Springer.

[33] Osisanwo F, Akinsola J, Awodele O, Hinmikaiye J, Olakanmi O, Akinjobi J (2017). Supervised machine learning algorithms: classification and comparison. Int J Comput Trends Technol (IJCTT)..

[34] du Plessis L, Skunca N, Dessimoz C (2011). The what, where, how and why of gene ontology–a primer for bioinformaticians. Brief Bioinform.

[35] Hess CT. Clinical Guide to Skin and Wound Care. 6th ed. 2008; Philadelphia, PA: Lippincott Williams & Wilkins.

[36] Hadian Y, Fregoso D, Nguyen C, Bagood MD, Dahle SE, Gareau MG, Isseroff RR (2020). Microbiome-skin-brain axis: a novel paradigm for cutaneous wounds. Wound Repair Regen..

[37] Gerber PA, Buhren BA, Schrumpf H, Homey B, Zlotnik A, Hevezi P (2014). The top skin-associated genes: a comparative analysis of human and mouse skin transcriptomes. Biol Chem.

[38] Zomer HD, Trentin AG (2018). Skin wound healing in humans and mice: challenges in translational research. J Dermatol Sci.

[39] Vázquez-Chona FR, Lu L, Williams RW, Geisert EE (2008). Genomic loci modulating the retinal transcriptome in wound healing. Gene Regul Syst Bio..

[40] Bagood MD, Gallegos AC, Medina Lopez AI, Pham VX, Yoon DJ, Fregoso DR, Yang HY, Murphy WJ, Isseroff RR (2021). Re-examining the paradigm of impaired healing in the aged murine excision wound model. J Invest Dermatol.

[41] Tusher VG, Tibshirani R, Chu G (2001). Significance analysis of microarrays applied to the ionizing radiation response. Proc Natl Acad Sci.

[42] Smyth GK. Limma: linear models for microarray data. In Bioinformatics and computational biology solutions using R and Bioconductor. 2005; pp. 397–420. Springer, New York, NY.

[43] Conesa A, Nueda MJ, Ferrer A, Talón M (2006). maSigPro: a method to identify significantly differential expression profiles in time-course microarray experiments. Bioinformatics.

[44] Nueda MJ, Tarazona S, Conesa A (2014). Next maSigPro: updating maSigPro bioconductor package for RNA-seq time series. Bioinformatics.

[45] Leng N, Li Y, McIntosh BE, Nguyen BK, Duffin B, Tian S (2015). EBSeq-HMM: a Bayesian approach for identifying gene-expression changes in ordered RNA-seq experiments. Bioinformatics.

[46] Äijö T, Butty V, Chen Z, Salo V, Tripathi S, Burge CB (2014). Methods for time series analysis of RNA-seq data with application to human Th17 cell differentiation. Bioinformatics.

