# Supplementary material for: Robust classification of wound healing stages in both mice and humans for acute and burn wounds based on transcriptomic data
Source: BMC Bioinformatics. 2023 Apr 25;24:166. doi: 10.1186/s12859-023-05295-z (PMC10127407; doi:10.1186/s12859-023-05295-z)
Supplement: Supplementary file 1 — Additional file 1. Supplementary S1: “Data selection”—analysis of existing wound transcriptomic datasets. Supplementary S2: “Fold change as an indicator of highly differentially expressed genes”—comparison of 3 main datasets under consideration. [file 12859_2023_5295_MOESM1_ESM.docx]

**Supplementary S1.**

**Data selection**


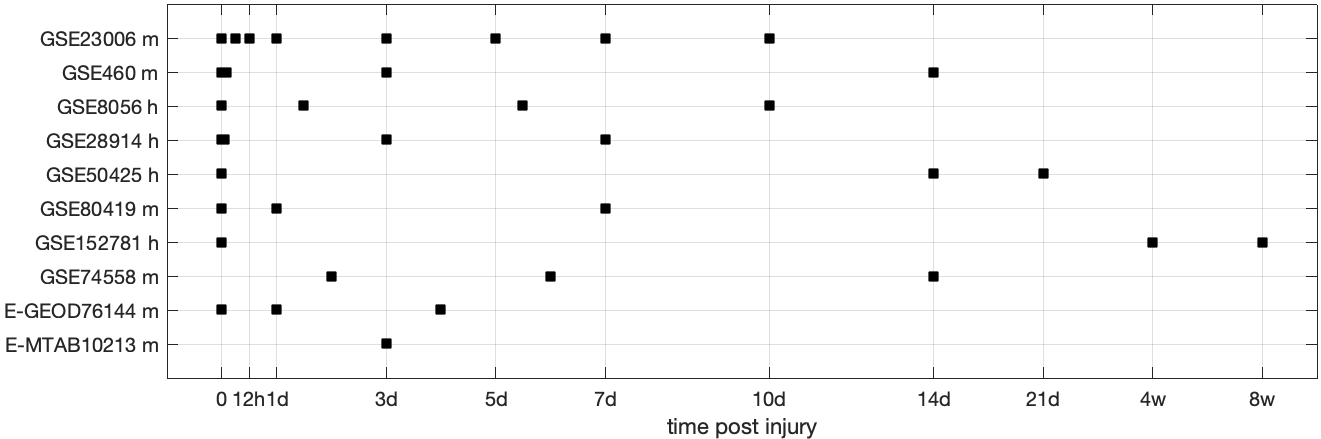


***Figure S1 Comparison of time points available in different wound transcriptomic datasets. Datasets titles are written on the left and marked as “m” or “h” corresponding to mouse or human.***

**Table S1 Information about several existing wound transcriptomics datasets.**

| Dataset ID, reference | Species | Wound type and size | Timepoints | Other information about dataset |
| --- | --- | --- | --- | --- |
| GSE23006, [Chen 2010] | Mouse | Surgical, 1mm | 0, 6h, 12h, 24h, 3d, 5d, 7d, 10d | Skin vs oral wound comparison. |
| GSE460 | Mouse | Burn, unknown size | 0, 2h, 3d, 14d |  |
| GSE8056, [Greco, 2010] | Human | Burn, different size | 0, 1-3d, 4-7d, >7d |  |
| GSE50425 | Human | split-thickness skin graft harvesting | 0, 14d, 21d |  |
| GSE28914, [Nuutila, 2012] | Human | split-thickness skin graft donor site | 0, 1h, 3d |  |
| GSE80419, [Kolumam, 2017] | Mouse | Surgical, 6mm | 0, 24h, 7d | 24h and 7d wound samples are from treated wounds |
| GSE152781, [Ud-Din, 2021] | Human | Surgical, 5mm | 0, week 4, week 8 | treatment by Vemurafenib vs placebo, data is presented as treatment to placebo ratio, |
| GSE74558, [Escuin-Ordinas, 2016] | Mouse | Full depth incision | 2d, 6d, 14d | with/without Vemurafenib treatment, no t=0 timepoint data. |
| GSE76144, [Kostarnoy, 2017] | Mouse | Surgical, 4 mm | 0, 1d, 4d |  |
| E-MTAB-10213, [Crompton, 2021] | Mouse | Laser microdissection incision | 3d | No t=0 timepoint, only one timepoint. |

**Supplementary S2.**

**Fold change as an indicator of**

**highly differentially expressed genes**

To be able to distinguish transcriptomic signatures associated with each wound healing stage, the expression profile across selected genes should be different at different stages. In other words, we are seeking genes with highly dynamic levels of expression across wound healing stages.

Suppose that a gene’s expression during wound healing is given by a vector of intensities: $\vec{g}=\left[ g\left( t_{0} \right),g\left( t_{1} \right),\cdots,g(t_{k}) \right]$ at time points $t_{0}, t_{1},\ldots,t_{k}$. Let the maximal and minimal levels of expression observed for a specific gene be: $I_{max}=max(g\left( t_{i} \right) )$, $I_{min}=min(g\left( t_{i} \right) )$. We define the relative maximum observed relative fold change as:

$$\Delta G=\frac{I_{max}-I_{min}}{I_{min}}$$

to account for variations in baseline expressions across datasets. To find common and unique features of the wounds considered, we try to differentiate genes that are commonly differentially expressed in all wounds from those with high variation across wounds.

Figure S2.1 shows the distribution of genes by the value of relative fold change $\Delta G$. As seen from the Figure, the distribution has one and the same unimodal shape in each dataset but with different means. We note there is no “natural” threshold value for $\Delta G$ to distinguish between genes highly differentially expressed across healing and non-differentially expressed. Furthermore, selecting the same threshold for all datasets would bias results and could significantly minimize the number of genes identified as highly expressed across all datasets with some data sets having an overall lower mean expression.


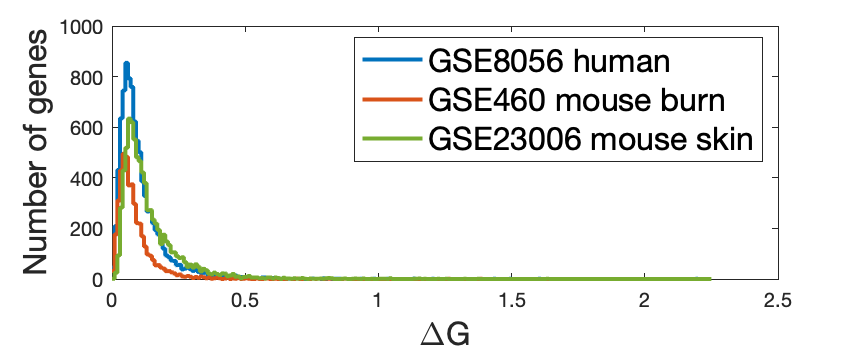


**Figure S2.1 Distribution of genes by the value of fold change** $\boldsymbol{\Delta}\boldsymbol{G}$ **in 3 datasets after filtering.**

Figure S2.2 demonstrates the relation of $\Delta G$ for each gene between datasets. Each gene is presented by one point with abscissa $\Delta G$ from one dataset and ordinate $\Delta G$ from another dataset. One can see that there is no strong correlation of $\Delta G$ between each pair of wounds with Spearman rank correlation coefficients calculated in Table S2.1.


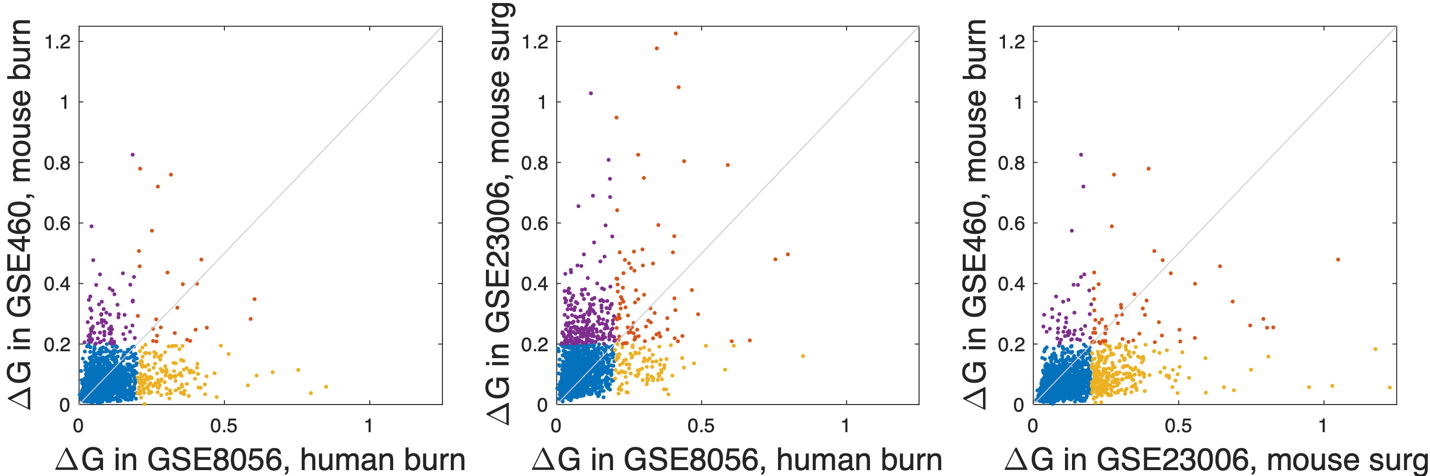


**Figure S2.2. Comparison of gene fold change** $\boldsymbol{\Delta}\boldsymbol{G}$ **between datasets for all intersecting genes. Each point corresponds to one of 1622 genes shared by all 3 datasets. Different colors denote genes with** $\boldsymbol{\Delta}\boldsymbol{G}$ **above (orange) or below (blue) 0.2 in both datasets as well as those with differing fold changes across dataset (purple, yellow).**

**Table S2. Spearman correlation coefficients between datasets calculated by different ranks.**

| Datasets:  Rank type | mouse surg vs  mouse burn | mouse surg vs  human | mouse burn vs  human |
| --- | --- | --- | --- |
| Fold change $\Delta G$ | 0.42 | 0.36 | 0.27 |
| Max upregulation $\Delta G_{u}$ | 0.39 | 0.34 | 0.22 |
| Max downregulation $\Delta G_{d}$ | 0.22 | 0.29 | 0.15 |

The distribution of $\Delta G$ is similar for all 3 datasets (Figure 1), however, the correlation coefficients between datasets are <0.5 (Table 4), i.e. if the gene has high/low $\Delta G$ in one wound, it does not necessarily imply highly changing dynamics in another wound (Figure S2.2).

**Supplementary S3.**

**Table of cluster genes and gene ontology analysis**

***Table S3 Gene clusters.***

**Analysis of the biological meaning of each clusters’ genes**

The number of genes in each cluster is too small for complete Gene Ontology analysis. However, to understand if clusters are related to wound healing stages, we uploaded gene list from each cluster to geneontology.org resource web page. Then GO-terms were arranged by p-value and first 10 GO terms were taken for each cluster. Then ten GO-terms from cluster were uploaded to QuickGO web resource and main branches of GO-tree containing those 10 terms are listed here.

Gene Ontology analysis of the biological meaning of each clusters’ genes shows following processes related to each of 5 clusters: (1) signaling, metabolic processes, localization, angiogenesis; (2) development, apoptosis, regulation of actin, regulation of relaxation, biosynthesis; (3) immune system process, inflammation, phagocytosis; (4) cell division and mitosis; (5) cell development, response to stimulus, regulation of proteins.

**Supplementary S4.**

**Wound Stage Prediction Model Results**


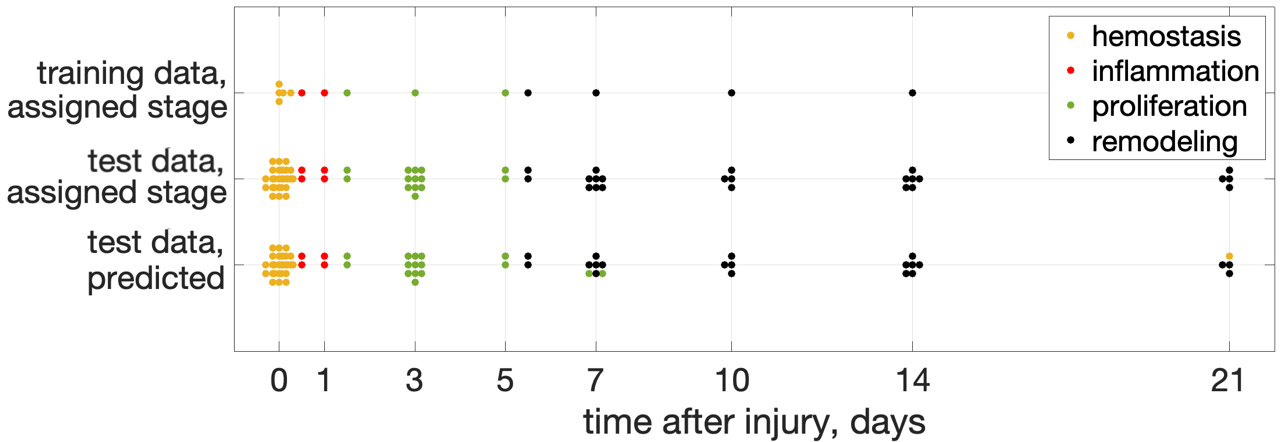


***Figure S4-1. Wound healing stage prediction results. The time axis is horizontal, and colored points correspond to the wound stages. Top row – training data, middle row – test data, stages were assigned based on timing. Bottom row – test data, model-predicted wound healing stages.***


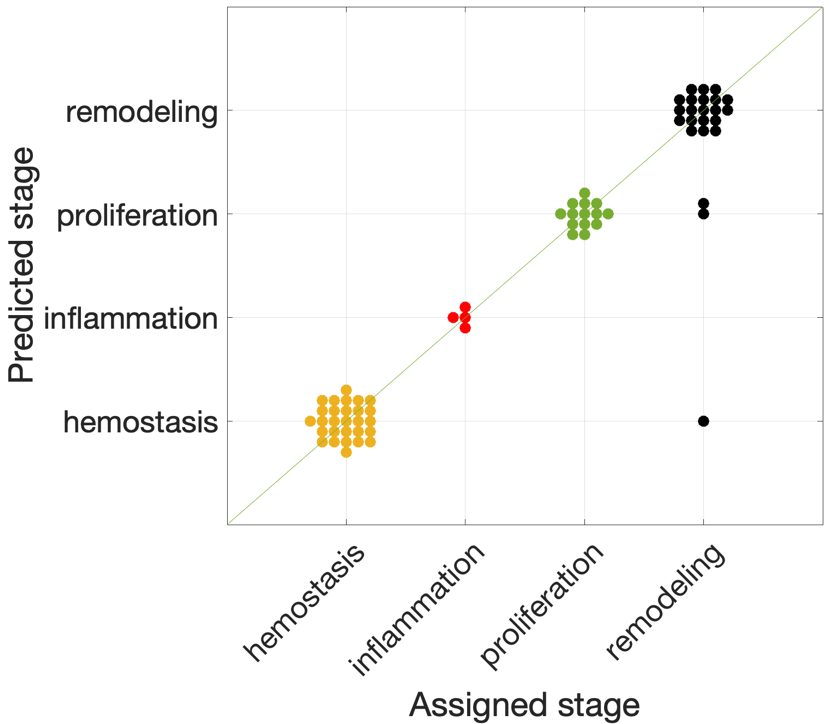


***Figure S4-2. Wound healing stage prediction results. Correspondence between assigned and predicted stages in the test data (replicates 2 and 3 from GSE23006, GSE460, GSE8056, and the full set of data points from GSE28914 and GSE50425). Colors indicate stages assigned to each data point (see legend in Figure S4-1).***
